# Factors for hesitancy towards vaccination against COVID-19 among the adult population in Puducherry, India – a cross sectional study

**DOI:** 10.1186/s12889-023-17095-4

**Published:** 2023-11-10

**Authors:** Raja Jeyapal Dinesh, Rajendran Dhanalakshmi, Priskilla Johnson Jency, Adinarayanan Srividya, Balakrishnan Vijayakumar, Ashwani Kumar

**Affiliations:** 1 Unit of Epidemiology & Operational Research, ICMR-VCRC, Indira Nagar, Puducherry, 605 006 India; 2 Unit of Biostatistics & VBD Modeling, ICMR-VCRC, Puducherry, India; 3 Director, ICMR-VCRC, Puducherry, India

**Keywords:** COVID-19, Pandemic, Vaccination, Vaccine Hesitancy

## Abstract

**Background:**

Vaccine hesitancy is a complex phenomenon that threatens global health. Present-day communication technology has paved the way for self-education but also contributed to the infodemic surrounding vaccination. This has resulted in pockets of people who are reluctant, refuse recommended vaccinations, or choose to delay being vaccinated. The present study was designed to estimate the magnitude of hesitancy towards the COVID-19 vaccination and determine its associated factors in the community.

**Methods:**

This cross-sectional study was conducted among 776 adults aged ≥ 18 years in 15 clusters in Puducherry district, India, between March 2022 and May 2022. Face-to-face interviews were conducted using a validated, structured questionnaire. Socio-demographic variables, co-morbidities, attitudes towards vaccination, etc., were expressed as frequencies and percentages. Vaccine hesitancy was dichotomized with the median score as the cut-off and reported as a proportion with a 95% confidence interval. Univariate and multivariate analyses were carried out to determine the factors associated with vaccine hesitancy.

**Results:**

The mean age of participants was 43.3 ± 14.8 years, with the majority being female (67.0%). Nearly 92.4%, 74.4%, and 0.5% of participants received their first, second, and precautionary doses, respectively, during the study period. Among the unvaccinated, 93.2% were unwilling to receive any dose of vaccination. More than half of the participants were hesitant towards vaccination, according to the vaccine hesitancy scale. Participants aged above 45 years were less hesitant, while those educated up to school level, belonging to the upper socio-economic class, never tested for COVID-19 in the past, and having a negative attitude towards vaccination were significantly associated with higher vaccine hesitancy.

**Conclusions:**

It is imperative to address vaccine hesitancy by alleviating existing fears and misconceptions in the community through efficient communication strategies to win the fight against current as well as future public health emergencies.

## Introduction

India, in its battle against the COVID-19 pandemic, launched one of the world’s largest vaccination drives on January 16, 2021, with two vaccines, viz., AstraZeneca-Covishield and the indigenously developed Covaxin, in a phased manner, with the initial beneficiaries being healthcare workers, frontline workers, people ≥ 50 years old, and those below 50 years old with comorbidities. Gradually, this was extended to include all individuals ≥ 45 years (April 1, 2021) and all adults ≥ 18 years by May 1, 2021, when the dreadful second wave was at its peak and more deaths were reported among those unvaccinated [1]. Emerging evidence supports the protective effect of vaccination against severe forms of disease and reduces hospitalization and mortality [2, 3]. The emergence of highly infectious *Omicron* variant, waning immunity, and the occurrence of breakthrough infections prompted the Government of India to recommend a precautionary (third) dose for adults and also introduce vaccination (Covaxin) for children aged 15–17 years around the first week of January 2022 [1]. Till date, the government has administered more than 2 billion vaccine doses successfully, free of cost, as per data available on CoWin, an online portal for registration for vaccination and real-time monitoring of doses administered across the country by the Government of India [4].

Despite the tremendous efforts by the government, several states were battling vaccine hesitancy among their people throughout the implementation period [5, 6]. According to the World Health Organization (WHO), vaccine hesitancy is one of the top ten threats to global health, especially during public health emergencies of international concern such as COVID-19. It defines vaccine hesitancy as the delay in acceptance or refusal of a safer vaccine despite the availability of vaccination services, often influenced by complacency, convenience, and confidence [7]. It is mostly driven by a lack of awareness, misinformation, and misplaced beliefs about vaccination among the masses [7]. Vaccine hesitancy poses a serious threat during pandemic situations by delaying the development of herd immunity as well as leading to an increase in disease morbidity and mortality, hindering the efforts of the government [8].

Studies have reported that mere availability of vaccines does not always result in acceptance owing to the interplay of several factors, such as age, gender, perceptions, knowledge, and beliefs, etc., across communities [9]. Hence, it is imperative to understand the role played by these factors to efficiently tackle vaccine hesitancy. A longitudinal study during the second wave between March and May 2021 in Puducherry, India, reported an increasing trend in vaccine hesitancy (32.7%; 4% increase) and refusals (35.6%; 10% increase) among the adult participants [10]. Another study in the neighboring state of Tamil Nadu (July 2021) among adults also documented higher levels of hesitancy (40.7%) and refusal (19.5%) [11]. This was attributed to waning community perceptions and confidence in vaccine efficacy and safety over time. Subsequently, the occurrence of milder clinical symptoms not requiring hospitalization during the third wave and reports on breakthrough infections post-vaccination established a sense of complacency and vaccine fatigue in the minds of people [12]. Studies have documented the perceived fears of the community over the adverse effects of vaccination, predominantly induced by the social media-related infodemic [13]. In order to successfully combat any outbreak, it is necessary to tackle misinformation, follow precautionary measures, and promote vaccination, if available, among the eligible and vulnerable populations, both now and during future outbreaks or pandemics. Therefore, this study was conducted to document the vaccination status and estimate vaccine hesitancy towards the COVID-19 vaccination and its associated factors, such as socio-demography, knowledge, perceptions, beliefs, etc., among the adult population in Puducherry, India.

## Methodology

### Study settings

The study was conducted in Puducherry district, the capital of the Union Territory of Puducherry and a coastal district in south India with a Census population of 12.3 lakhs and a literacy rate of 85.4% [14]. In September 2021, this district had documented vaccine hesitancy as high as 40%, with relatively low coverage among people over 60 who were more vulnerable. This was despite the mass vaccination festivals, intensive door-to-door vaccination, and mass media campaigns carried out by the local government [15]. Initially, in January 2021, only Covishield was introduced in this district, and Covaxin was made available later, in January 2022.

### Study design and period

This community-based cross-sectional study was conducted among adults aged ≥ 18 years in Puducherry district between March 2022 and May 2022.

### Sample size

Based on the available estimate of vaccine hesitancy [10] from the literature (25.6%), at a 5% level of significance allowing a 7% margin of error, a non-response rate of 10%, and a design effect of 1.5% (considering homogeneity of responses due to clustering within the study areas), the minimum sample size was calculated as 250. Therefore, it was decided to sample a minimum of 250 individuals each from the urban, peri-urban (these are fringe areas of cities or adjoining rural areas, which are intrinsically linked with the city economy, experience constant transformation, and are characterized by a mix of rural and urban activities [16]) and rural areas of Puducherry. The final sample size was 750.

### Sampling method

**Fig. 1:**
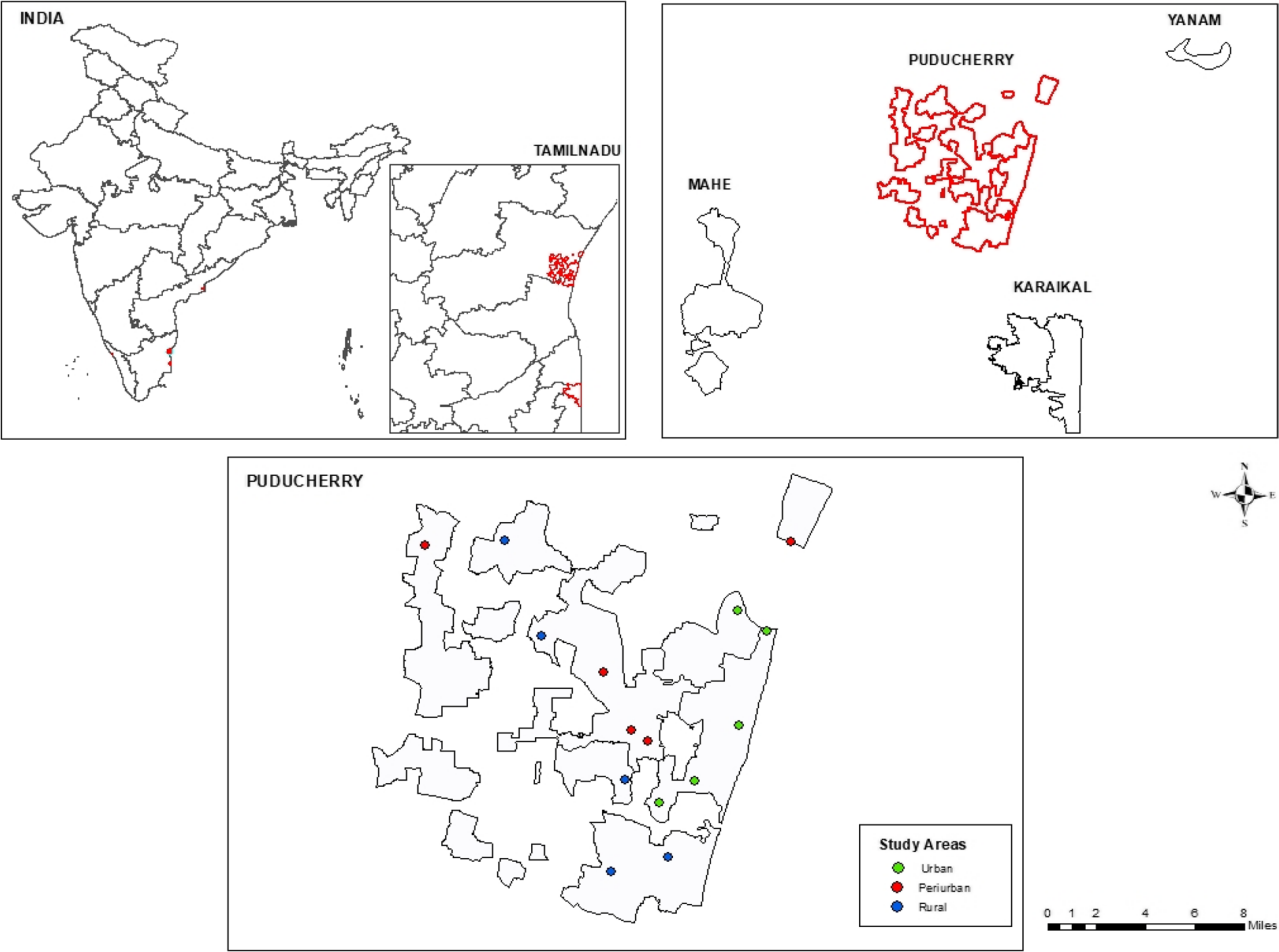
Map showing study areas in Puducherry district

**Fig. 2:**
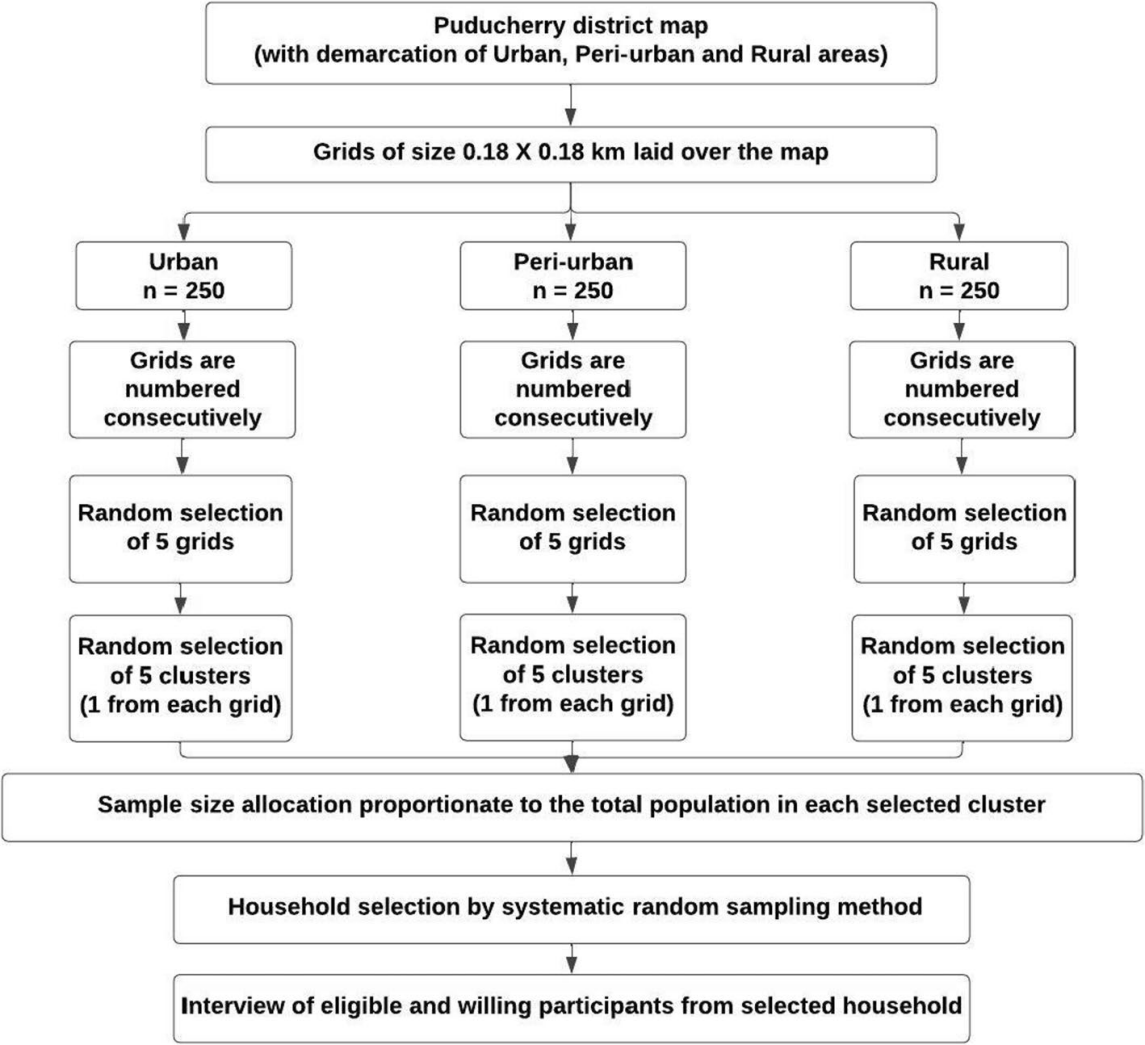
Sampling method

The study was conducted in 15 clusters, with 5 each representing the urban, peri-urban, and rural areas in Puducherry district (Fig. 1). The study sites were selected based on the grid-sampling method. Grids of size 0.18 km × 0.18 km were overlaid on the entire district of Puducherry with clearly defined urban, peri-urban, and rural areas. The grids were numbered consecutively, and 15 grids were randomly selected. Of these, five each were randomly allocated to urban, peri-urban, and rural areas. From each allocated grid, one area was randomly selected for the study. Likewise, 15 clusters were selected to represent five clusters each from urban, peri-urban, and rural areas. The sample size of 250 was proportionately allocated to the five randomly selected clusters based on the Census population. The sampling method is depicted in (Fig. 2).

### Study tool

A structured questionnaire was developed by the authors following a comprehensive literature review, and the content was verified by a panel of experts. The questionnaire collected details on socio-demography, smoking/alcohol status, co-morbid diseases, past history of (h/o) testing for COVID-19, vaccination status, vaccine type, vaccine hesitancy, and its reasons from study participants. Vaccine hesitancy was assessed using a pre-validated Vaccine Hesitancy Scale (VHS) adopted from the WHO SAGE (Strategic Advisory Group of Experts on Immunization) Working Group on Vaccine Hesitancy [7, 17]. The VHS scale consisted of 14 items measured on a 5-point Likert scale ranging from ‘strongly disagree’ to ‘strongly agree’ with ‘neither agree’, ‘nor disagree’ as a midpoint. The total score ranged from 14 to 70. The median score was considered the cut-off point, and scores above the median indicated higher vaccine hesitancy [18]. The questionnaire was prepared in the local language, Tamil. Translations and back-translations were carried out by language experts. The questionnaire was pilot tested among 20 individuals in a locality different from the study area prior to the commencement of the study. These results were not included in the final analysis. The homogeneity of the question items was evaluated using Cronbach’s alpha coefficient and was found to be 0.864 (Cronbach’s alpha > 0.80 suggests good internal consistency).

### Data collection methods

A trained team consisting of one medical doctor and one social worker was involved in the data collection. A systematic random sampling method was used for the selection of households in the identified areas. All adults aged ≥ 18 years who were physically available at the time of visit and volunteered to participate in the study were interviewed using the structured questionnaire. The social worker established a good rapport with the participants by clearly explaining the purpose of the study. They were assured that their personal information provided will be used only for research purposes and will not be disclosed to anyone and anonymity will always be maintained. A written informed consent was obtained from all interested participants prior to the personal interview. Temporary residents (with less than one year of stay) and those unwilling to participate were excluded. Vaccination certificates, if available, were verified to ensure data reliability. In the absence of a certificate, the status was verified on the CoWIN portal using their registered mobile number. On completion of the interview, the team informed the unvaccinated individuals about the importance of vaccination, allayed their fears, and encouraged them to get vaccinated as soon as possible.

### Statistical analysis

Data analysis was performed using Statistical Package for Social Sciences v21.0 (IBM SPSS Statistics Inc., Armonk, NY, USA) and Stata v17.0 (StataCorp, College Station, TX). Continuous variables such as age were expressed as mean ± standard deviation and the vaccine hesitancy score as median and interquartile range (IQR). The categorical variables were summarized as frequencies and percentages. Vaccine hesitancy (VH, dependent variable) was interpreted as a proportion with a 95% confidence interval. The association between VH and other independent variables (IVs) was performed using the univariate χ^2^ test. Subsequently, a multivariate logistic regression model with and without random effects (for area-level effects) was fitted with those IVs with a P value < 0.20 in univariate analysis. The likelihood ratio test (LR test) was used to assess the best model, which was finally used to identify the significant factors (with a P value < 0.05) associated with vaccine hesitancy among the adults.

### Ethical considerations

This study was approved by the Institute Human Ethics Committee (IHEC/0422/N/F, dated February 25, 2022). The research team described the study clearly in the local language, and written informed consent was obtained from all willing adult participants aged 18 years and older. The study was explained clearly to illiterate participants in the local language, and written informed consent was obtained from a literate witness available at the time of survey. All methods were carried out in accordance with relevant guidelines and regulations in the Declaration of Helsinki. Prior permission was obtained from the local health authorities for conducting the study. The privacy and confidentiality of the responses obtained were ensured. STROBE guidelines for reporting cross-sectional studies were followed.

## Results

### Socio-demographic details

A total of 776 adults from 466 households (minimum one from each household) from urban, peri-urban, and rural areas of Puducherry district participated in the study. The mean age of study participants was 43.3 ± 14.8 years. The majority of 520 (67.0%) were female, and the rest were male. The sociodemographic characteristics of the study participants are summarized in Table 1.

**Table 1:** Socio-demographic characteristics of study participants (*N* = 776)

Socio-demographic characteristics	n (%)
Area of residence	
Urban	264 (34.0)
Peri-urban	258 (33.3)
Rural	254 (32.7)
Age class (years)	
18 – 45	442 (56.9)
46 – 60	228 (29.4)
> 60	106 (13.7)
Gender	
Male	256 (33.0)
Female	520 (67.0)
Education	
Illiterate	90 (11.6)
Up to Secondary school	499 (64.3)
Graduate & above	187 (24.1)
Employment status	
Unemployed	474 (61.1)
Employed	302 (38.9)
Socioeconomic status [18]	
Class I & II	565 (72.8)
Class III-V	211 (27.2)
Religion	
Hindu	756 (97.4)
Others	20 (2.6)
Marital status	
Married	632 (81.4)
Unmarried	91 (11.8)
Others	53 (6.8)
Family type	
Joint	271 (34.9)
Nuclear	505 (65.1)

#### Self-reported personal behaviour and co-morbidities

Only 29 (3.7%) participants reported either having the habit of smoking and/or consumption of alcohol. About 208 (26.8%) participants reported having co-morbidities. Hypertension 112 (38.2%) and diabetes 102 (34.8%) were the most commonly self-reported comorbidities, followed by hypothyroidism 17 (5.8%), asthma 15 (5.1%), osteoarthritis 8 (2.7%), cardio-vascular diseases 8 (2.7%), epilepsy 3 (1.0%), anemia 4 (1.4%), and several others.

#### Past history of testing for COVID-19

Nearly 353 (45.5%) participants were unaware of any past exposure and never got themselves tested for COVID-19 during the pandemic. The remaining 423 (54.5%) reported having undergone either PCR or RAT testing for COVID-19 at least once in the past. Among them, 98 (23.2%) had tested positive and were isolated and treated based on the severity of symptoms.

#### Attitude towards COVID-19 vaccination

Nearly 490 (63.1%) affirmed that vaccination against COVID-19 would protect them from severe forms of disease, while the rest 286 (37.0%) either denied or had no opinion. The majority of participants, 661 (85.2%), agreed it is necessary to practice COVID-19-appropriate behavior such as wearing a mask, hand washing, and maintaining social distance in public places even after vaccination. Nearly 533 (69.0%) accepted that it is important to vaccinate children < 18 years if a vaccine is available.

#### COVID-19 vaccination status

**Fig. 3:**
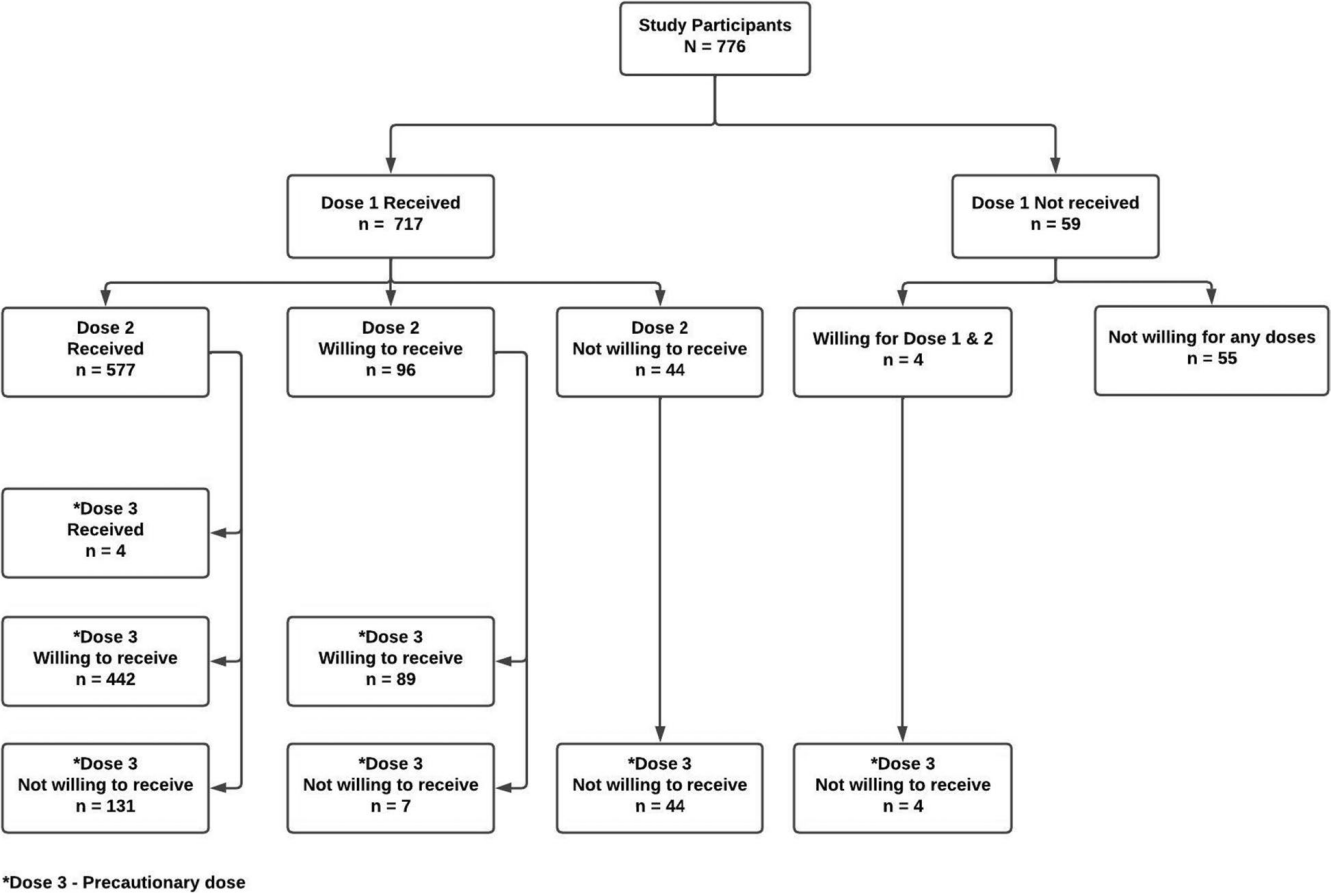
COVID-19 vaccination status and willingness for vaccination

Among the study participants (*N* = 776), 717 (92.4%), 577 (74.4%), and 4 (0.5%) received their first, second, and precautionary doses of the COVID-19 vaccine, respectively, during the study period. Of them, 140 participants were due for the second dose and 573 for the precautionary dose (Fig. 3). Most participants, 702 (98.0%), received the vaccine Covishield, and very few received Covaxin. Among those who did not receive any dose of vaccine, 59 (7.6%), 14 did not receive vaccination as they had comorbid conditions such as diabetes, hypertension, asthma, cancer, epilepsy, hyperuricemia, etc., and another four were either pregnant or lactating mothers. The remaining cited reasons (multiple responses) like: fear of adverse events 24 (38.7%), no need for vaccination as there is no risk of disease 15 (24.2%), fear of needles 10 (16.1%), fear of adverse effects owing to their habit of smoking cigarettes and/or drinking alcohol 5 (8.1%), belief in naturally acquired immunity 4 (6.5%), being busy with other work 3 (4.8%), and for religious reasons 1 (1.6%).

#### Willingness for vaccination

Only four of the unvaccinated (those who did not receive any dose of vaccine) were willing to get vaccinated in the near future. Among those who were yet to receive the second dose (*n* = 140), 44 (31.4%) and 51 (36.4%) were unwilling to receive the second and precautionary doses, respectively. Whereas among those eligible for the precautionary dose (*n* = 573), 131 (22.9%) were unwilling (Fig. 3). The most common reasons cited for not receiving second and precautionary doses were fear of adverse events 106 (42.9%), no need for further vaccination as there is no risk of disease 49 (19.8%), one dose will be sufficient 37 (14.9%), vaccines being less effective 13 (5.3%), and perception of natural immunity being better than acquired immunity offered by vaccines 4 (1.6%), amongst several others.

#### Vaccine hesitancy (assessed by the VHS scale) and its determinants

The median VHS score of study participants was 37 (IQR: 31–42). Considering the proportion of participants who scored above the median cutoff point, 51.5% (95% CI 47.9–55.2) of study participants had a higher hesitancy towards the COVID-19 vaccination. On univariate analysis, age, education, socio-economic status, marital status, family type, comorbidities, and attitude towards vaccination were significantly associated with vaccine hesitancy (*P* < 0.05).

As mentioned earlier, both random and fixed effects LR models were fit to find the factors associated with vaccine hesitancy among the adults. However, as there were no area-level effects as ascertained based on the LR test (*P* = 0.45) comparing the two regression models, the naïve LR model was considered to determine the association between various independent variables and vaccine hesitancy.

Participants aged above 45 years were less hesitant towards vaccination compared to the younger age class (18–45 years). Those who had school-level education and belonged to a higher socio-economic class were 2.1 and 1.7 times more vaccine hesitant (*P* < 0.05) compared to the illiterate and lower socio-economic class respectively. Participants who never tested for COVID-19 in the past and had negative beliefs such as vaccination will not protect from severe disease and felt it was not necessary to follow COVID-19-appropriate behavior post-vaccination were found to be 1.7, 2.0, and 1.9 times more hesitant for vaccination compared to their counterparts. Also, those who felt children should not be vaccinated or had no opinion were 2.2 times more likely to be vaccine hesitant (Table 2).

**Table 2:** Factors associated with vaccine hesitancy among the adults in Puducherry district (*N* = 776)

Variables	n	Vaccine Hesitancy n (%)	Univariate analysis		Multivariate analysis	
			Unadjusted OR (95% CI)	*P*-value	Adjusted OR (95% CI)	*P*-value
***Area type***						
Urban	264	129 (48.9)	1		Not included for the analysis	
Peri-urban	258	139 (53.9)	1.22 (0.87–1.72)	0.511		
Rural	254	132 (52.0)	1.13 (0.80–1.60)			
***Age class (years)***						
18–45	442	256 (57.9)	1		1	
**46–60**	228	102 (44.7)	0.59 (0.43–0.82)	< 0.001	0.64 (0.43–0.95)	0.026*
**> 60**	106	42 (39.6)	0.48 (0.31–0.75)		0.57 (0.33–0.99)	0.044*
***Gender***					Not included for the analysis	
Male	256	129 (50.4)	1	0.651		
Female	520	271 (52.1)	1.07 (0.79–1.46)			
***Education***						
Illiterate	90	31 (34.4)	1	0.001	1	
**Up to Secondary school**	499	262 (52.5)	2.10 (1.28–3.48)		2.11 (1.22–3.66)	0.007*
> Graduate	187	107 (57.2)	2.55 (1.46–4.45)		1.88 (0.97–3.65)	0.063*
***Employment status***						
Unemployed	474	239 (50.4)	1	0.432	Not included for the analysis	
Employed	302	161 (53.3)	1.12 (0.83–1.52)			
***Socioeconomic status*** [19]						
Class III-V	211	96 (45.5)	1	0.040	1	
**Class I & II**	565	304 (53.8)	1.39 (1.02–1.92)		1.67 (1.16–2.41)	0.006*
***Religion***						
Hindu	756	387 (51.2)	1	0.216	Not included for the analysis	
Others	20	13 (65.0)	1.77 (0.70–4.49)			
***Marital status***						
Married	632	328 (51.9)	1		1	
Unmarried	91	54 (59.3)	1.35 (0.85–2.18)	0.014	1.08 (0.63–1.85)	0.770
Others	53	18 (34.0)	0.48 (0.25–0.89)		0.79 (0.38–1.6)	0.507
***Family type***						
Joint	271	126 (46.5)	1	0.045	1	0.203
Nuclear	505	274 (54.3)	1.37 (1.01–1.85)		1.32 (0.95–1.83)	
***Personal habit (smoking/alcohol)***						
Yes	29	19 (65.5)	1			
No	747	381 (51.0)	1.83 (0.84–3.98)	0.125	0.57 (0.24–1.35)	0.460
***Comorbidities***						
Yes	208	87 (41.8)	1	0.001	1	0.372
No	568	313 (55.1)	1.71 (1.24–2.35)		1.15 (0.79–1.69)	
***Past h/o of testing for COVID-19***						
Tested	423	206 (48.7)	1	0.082	1	0.001*
**Never tested**	353	194 (55.0)	1.29 (0.97–1.71)		1.71 (1.23–2.37)	
***Vaccine type (n* = *717)***				0.73		
Covaxin	15	7 (46.7)	1		Not included for the analysis	
Covishield	702	359 (51.1)	1.2 (0.4–3.3)			
***Vaccine protects from severe disease***						
Yes	490	226 (46.1)	1	< 0.001	1	
**No**	161	101(62.7)	1.96 (1.34–2.89)		2.01 (1.29–3.11)	0.002*
No opinion	125	73 (58.4)	1.63 (1.08–2.49)		1.59 (1.00–2.54)	0.05
*Necessary to practice COVID-19 appropriate behaviour post-vaccination:*						
Yes	661	320 (48.4)	1		1	
**No**	88	62 (70.5)	2.54 (1.5–4.29)	< 0.001	1.88 (1.1–3.21)	0.021*
No opinion	27	18 (66.7)	2.13 (0.89–5.46)		1.74 (0.68–4.47)	0.248
***Necessary to vaccinate children***						
Yes	533	237 (44.5)	1			
**No**	143	99 (69.2)	2.81 (1.87–4.27)	0.004	2.24 (1.43–3.51)	0.0001*
**No opinion**	100	64 (64.0)	2.22 (1.40–3.56)		2.23 (1.33–3.72)	0.002*

^*^Significant at 5% level

## Discussion

Herd immunity through vaccination is vital to combat and win over COVID-19. A study in the Brazilian Amazon, where COVID-19 was allowed to spread unmitigated among its population, documented that herd immunity could not be achieved even after 76% of its population were naturally infected with SARS-CoV-2. It concluded that mere exposure to natural infection will not aid in pandemic mitigation due to the existence of a robust neutralizing antibody response post-infection [20]. Immunization is an indispensable public health tool that saves millions of lives from life-threatening diseases every year. Despite the availability of scientifically proven and clinically tested vaccines for COVID-19, vaccine hesitancy remains an important public health challenge that needs to be addressed to bolster protective immunity in the community.

In the present study, about 92% and nearly three-quarters of participants had received their first and second doses of vaccination, respectively. However, more than a quarter were unwilling to take the precautionary dose. A study in Malaysia found a similar level of unwillingness (26.7%) towards the precautionary dose [21]. Another longitudinal study during the second wave of the pandemic in Puducherry reported an increasing trend in vaccination refusal (10% increase) among its study participants [10]. Evidence emerged that protection against hospitalization and severe COVID-19 disease wanes slowly after a two-dose schedule of any COVID-19 vaccine. With the emergence of highly infectious *Omicron* variant and other variants of concern, waning immunity and the occurrence of breakthrough infections prompted the Indian government to recommend a precautionary dose for adults. A systematic review of 27 studies also affirmed the need, efficacy, and effectiveness of booster (precautionary) dose vaccination against COVID-19 variants [22]. The authors concluded that precautionary dose vaccines should be made available, at least to those who are immunocompromised, have concomitant comorbidities, and are vulnerable [22]. Hence, it is imperative to create awareness among the masses about the importance of receiving a precautionary dose. Concern over serious reactions following vaccination was unfathomably the commonest reason cited in the present study and elsewhere [10, 23–27]. Perceptions such as a lack of need for primary vaccination and subsequent doses were also reported. These need to be strongly tackled with intense information, education, and communication strategies that allay the fears and concerns about vaccination existing among the people. Despite being implemented since January 2022, only 33% of the population had received the precautionary dose in Puducherry until May 2023 [28]. Notably, among the unvaccinated, more than 90% were unwilling to receive any vaccination at all, which is of serious concern and needs to be dealt with ardently. More than half of these unvaccinated were young, aged 18–44 years. Although few unvaccinated individuals reported to have comorbidities or were either pregnant or lactating mothers and unwilling to receive vaccination, citing these reasons, these were not absolute contraindications as such for vaccination. This further necessitates the need for robust awareness campaigns to dispel their fears. Several studies have reported more refusals towards vaccination among smokers and those who consume alcohol [29]. In the present study, individuals who had the habit of smoking and drinking alcohol were hesitant to get vaccinated owing to fear of adverse effects, which needed to be dispelled. The local government had undertaken several measures to allay the fears of people, dispel myths, and encourage vaccination among its people through awareness messages and conducted mass vaccination drives, including house-to-house campaigns, which resulted in high coverage with the first dose in the district [15].

More than half the participants had hesitancy towards vaccination (assessed by VHS scale), much higher than that previously reported (32.7%) [10]. The elderly, aged above 60 years and those above 45 years, were less hesitant towards vaccination, and this corroborates findings from other studies [10, 23]. This may be due to the early availability of vaccines for those over 45 years, better awareness regarding vaccination, or the fear of serious complications of the life-threatening disease making them more vulnerable [30]. Socio-demographic factors such as high socio-economic status and being educated up to secondary school level were significantly associated with higher vaccine hesitancy, which was in concurrence with the available literature [23, 27]. Exposure to the infodemic regarding vaccination on social media might have negatively influenced their intention towards vaccination. [13, 31]. Several studies had reported gender to be a significant predictor of vaccine hesitancy; however, no such association was found in the present study [23, 32–34]. Consistent with previous studies, we found higher vaccine hesitancy among those who had never tested for COVID-19 in the past, probably due to a lack of perception of the threat from the disease [24, 27, 31]. Another study reported that people with known comorbid conditions were less hesitant towards vaccination, probably due to better awareness about the increased severity of disease among them if they were unvaccinated [23]. However, such an association could not be elicited in the present study. Furthermore, participants with negative beliefs or attitudes towards vaccination (vaccine does not protect against severe disease, it is not necessary to practice COVID-19-appropriate behaviour, and it is not essential to vaccinate children) were significantly associated with higher vaccine hesitancy in the current study. It is more likely that these individuals may not vaccinate their children in the future due to their negative beliefs. Several contributory factors can be attributed to the existence of such negative beliefs in the community. With advances in communication technology and the ease of availability of gadgets, more people have easy access to the internet and social media. While it’s a boon at one end of the spectrum, as it immensely paves the way for self-education among the masses, there lurks the threat of sharing and believing in a lot of unscientific misinformation being circulated on social media. Evidence also supports the importance of tackling infodemics during pandemic situations [13]. This mandates the government and researchers to timely convey to the people accurate and factual information about scientific developments on COVID-19 vaccination in a simple and understandable manner through highly efficient communication strategies. It is also the responsibility of the community to avoid sharing unverified false information through social media that might trigger false beliefs, thereby hampering the ongoing efforts to mitigate the pandemic. These measures will enable the building of public confidence in vaccination not only during the current pandemic but also during similar public health emergencies in the future.

### Strengths and limitations

This community-based study was conducted using a validated questionnaire following a robust methodology. The vaccination status of study participants was verified using the CoWIN app using their registered mobile number if vaccination certificates were not physically produced at the time of the survey. There are a few limitations as well. The data collection was carried out during working hours, resulting in the inclusion of more female participants in the study. Also, the survey was conducted during a period when the precautionary dose was available only for healthcare/frontline workers and those with co-morbidities. With the availability of vaccines for everyone and new information on the efficacy and safety of precautionary dose, this could have influenced their intention to get vaccinated in the future. However, despite all efforts, the coverage of precautionary dose is still low (33%) in the district, as per recently available records [28]. The temporal association of causal factors could not be established, an inherent limitation of cross-sectional studies.

## Conclusions

Our study found that nearly three-fourths were vaccinated with the first and second doses of the COVID-19 vaccine, which reflects the untiring efforts by the government machinery. However, it also documented increasing vaccine hesitancy towards precautionary dose, the rate of which still remains low as per government records. It is imperative to make people aware of the precautionary dose of the COVID-19 vaccine. Although the number of new cases has drastically reduced, most COVID-19-related hospital admissions and deaths are being reported among those unvaccinated, as per treating physicians (unpublished). With several mutations being reported, uncertainty prevails when an outbreak with a new variant might occur. Vaccine hesitancy, if not addressed early, may not be limited to the pandemic vaccines but may continue to extend to other recommended vaccines as well. It is imperative to enhance and reinvigorate the importance of vaccination and alleviate fears among the general public to successfully combat any lurking disease outbreak in the future. Additionally, COVID-19-appropriate behaviour, such as the wearing of masks, the practice of social distancing, hand hygiene, cough etiquette, etc., should be promoted among the masses to curtail not only COVID-19 but any future pandemics.

## Data Availability

The datasets generated and analyzed during the current study are available upon reasonable request with due permission from the competent authority (The Director, ICMR-VCRC, Medical complex, Indira Nagar, Puducherry-605006, Tel: + 91–413-2,272,396) who can be contacted via email: director.vcrc@icmr.gov.in.
